# Renal effects of short‐term ketone monoester supplementation in healthy adults: A randomized, placebo‐controlled study

**DOI:** 10.14814/phy2.70829

**Published:** 2026-03-16

**Authors:** Trine Z. Lyksholm, Camilla L. Duus, Steffen F. Nielsen, Henrik H. Thomsen, Jesper N. Bech

**Affiliations:** ^1^ University Clinic in Nephrology and Hypertension, Gødstrup Hospital Herning Denmark; ^2^ Department of Clinical Medicine Aarhus University Aarhus Denmark; ^3^ Department of Renal Medicine Aarhus University Hospital Aarhus Denmark; ^4^ Medical Diagnostic Center University Clinic for Innovative Patient Pathways, Regional Hospital Central Jutland Viborg Denmark

**Keywords:** exogenous ketosis, glomerular filtration rate, natriuresis, potassium excretion, renal physiology

## Abstract

Recent studies suggest potential benefits of ketosis. However, research is lacking on its effects on renal physiology. This study aimed to investigate the renal effects of short‐term exogenous ketosis in healthy adults, focusing on glomerular function and electrolyte handling. In a double‐blind, placebo‐controlled, randomized crossover trial, 15 healthy participants received ketone monoester (KME) or placebo three times daily for 5 days under a standardized diet, separated by a 2‐week washout. On the fifth day of each intervention, participants collected a 24‐h urine sample. The following day, glomerular filtration rate (GFR) was assessed with ^99m^Technetium‐diethylene‐triamine‐pentaacetate (Tc‐99‐DTPA) clearance, and urinary electrolyte excretion was measured. Thirteen participants completed both treatment periods. Compared with placebo, KME treatment acutely increased GFR by 6.0 mL/min (*p* = 0.002) and sodium excretion increased by 69 μmol/min (*p* < 0.0001), while potassium excretion decreased (−31 μmol/min; *p* < 0.0001). Electrolyte excretion measured in 24‐h urine remained unchanged. In conclusion, short‐term KME supplementation was associated with acute increases in measured GFR and urinary sodium excretion, while reducing urinary potassium excretion in healthy adults. No changes in electrolyte excretion were detectable in the 24‐h urine, indicating that renal effects are not sustained over the full collection period.

## Graphical abstract

Randomized, double‐blind, placebo‐controlled crossover study examining the renal effects of five days of ketone monoester supplementation in healthy participants. Exogenous ketosis increased glomerular filtration rate (GFR) and fractional sodium excretion (FENa) during the examination day.

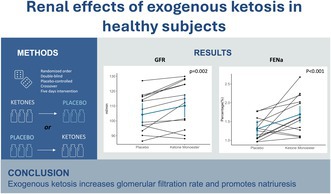

## 1 INTRODUCTION

Ketosis occurs as a normal physiological state during total fasting or carbohydrate restriction, also known as the ketogenic diet. Recently, intermittent fasting and ketogenic diets have become increasingly popular, partly due to the induction of endogenous ketosis. Ketone bodies are synthesized in the liver through the oxidation of free fatty acids when glycogen stores are depleted, serving as an alternative energy source during fasting (Palmer & Clegg, 2021). Ketone bodies are commercially available as a nutritional supplement often as ketone salts (KS) or a ketone ester (KE). Ingestion of these rapidly induces ketosis (Falkenhain et al., 2023).

Ketosis has previously been negatively associated with dysregulated type 1 diabetes mellitus. However, it has become clear that ketosis is associated with several beneficial effects. For example, studies suggest that ketone bodies may play a neuroprotective role in Parkinson's and Alzheimer's disease (Kashiwaya et al., 2000; Norwitz et al., 2020). Ketogenic diets have also been used in the management of refractory epilepsy in children (Zarnowska, 2020). Other reported benefits include improved exercise capacity, and ketone body infusion has recently shown to improve cardiac function in patients with heart failure (Gormsen et al., 2017; Yao et al., 2021).

Research on ketosis and renal physiology remains limited. Most available evidence derives from animal studies, which suggest that ketosis may confer renoprotective effects through anti‐inflammatory and anti‐fibrotic mechanisms (Fang et al., 2021; Tomita et al., 2020; Yao et al., 2021). More recently, clinical studies of ketogenic dietary interventions, including a nutritional programme incorporating adjunctive BHB‐citrate supplementation, have reported promising kidney‐related outcomes in patients with autosomal dominant polycystic kidney disease (ADPKD) or type 2 diabetes (Athinarayanan et al., 2025; Cukoski et al., 2023; Muensterman et al., 2025). However, most of the current human evidence is based on dietary approaches, and studies examining the renal effects of isolated exogenous ketosis are largely restricted to acute infusion experiments reporting increases in GFR without detailed assessment of urinary electrolyte handling (Fioretto et al., 1987). Hence, we conducted a randomized order, double‐blind, placebo‐controlled, crossover study with the aim to examine the effects of oral ketone supplementation on glomerular filtration, renal sodium and water balance, and the hormonal regulation of tubular transport mechanisms under standardized dietary conditions. We hypothesized that exogenous ketosis would enhance GFR and promote sodium excretion. A comprehensive understanding of the collective physiological effects of ketosis is crucial for assessing its potential therapeutic applications.

## 2 MATERIALS AND METHODS

### 2.1 Participants

Healthy males and females, aged 18–30, with BMI 18–30 kg/m^2^, safe anti‐conception, and with no prior history of chronic diseases were recruited by advertisement on social media. Exclusion criteria were diabetes, kidney, liver, or heart disease, cancer, alcohol or drug abuse, pregnancy or breastfeeding, and intermittent fasting or adherence to a ketogenic diet within 4 weeks before inclusion. Also, participants were prohibited from taking any prescription drugs other than contraceptives. All participants gave oral and written signed consent. The study was carried out at the University Clinic in Nephrology and Hypertension, Gødstrup Hospital, Denmark.

### 2.2 Study design and experimental setup

**FIGURE 1:**
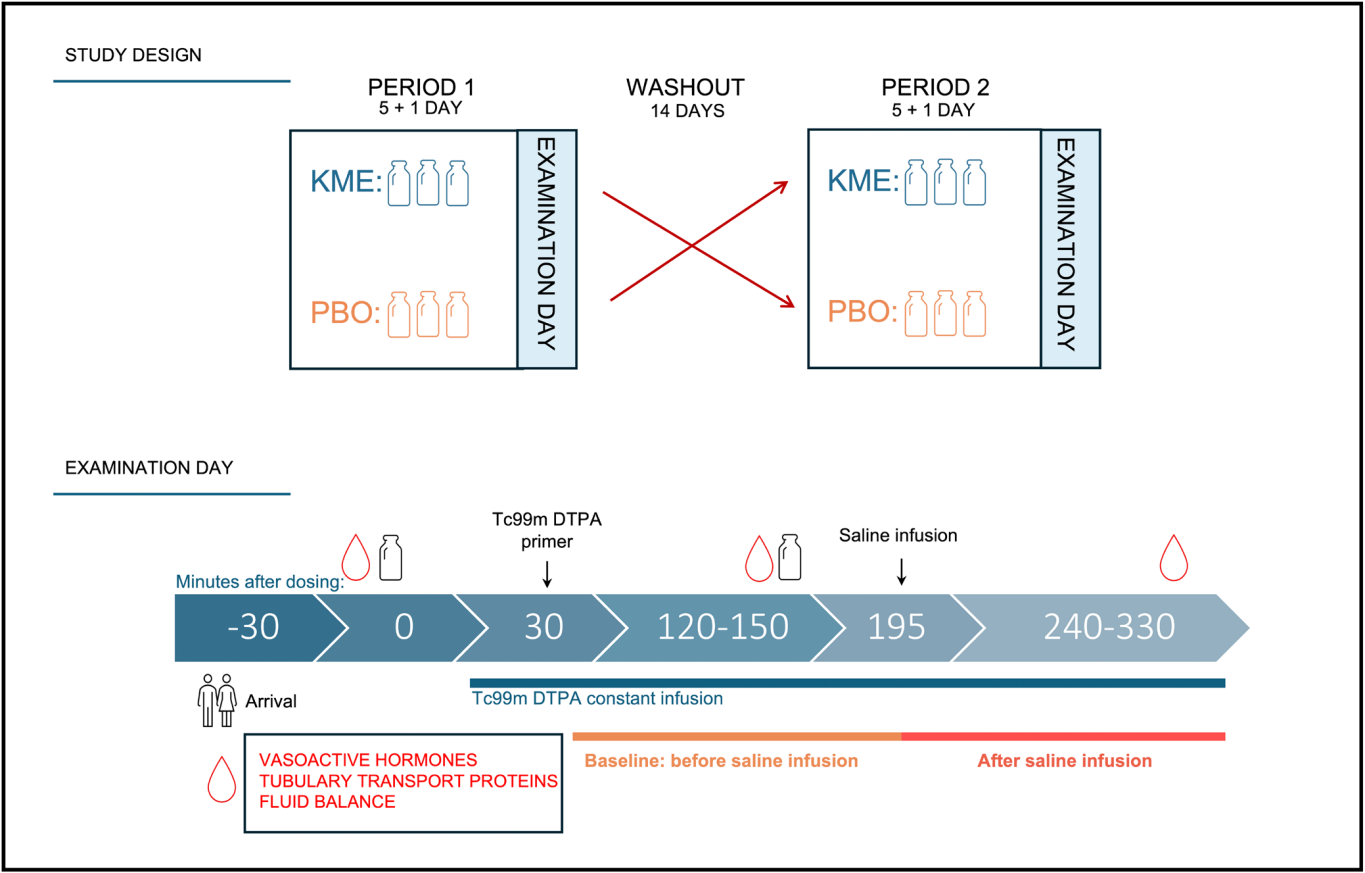
Study design and examination day. This randomized, double‐blind, crossover trial comprised two 5‐day intervention periods during which participants received ketone monoester (*KME*) or a taste‐ and volume‐matched placebo (*PBO*) three times daily under a standardized diet. On day 5, participants collected a 24‐h urine sample and subsequently attended an examination visit after an overnight fast. On the examination day, participants received *KME/PBO* at time 0 and again at 150 min. Glomerular filtration rate (*GFR*) was measured by ^99m^Technetium‐diethylene‐triamine‐pentaacetate (Tc‐99 m‐DTPA) constant infusion, initiated 30 min after the first dose. GFR, diuresis, and urinary electrolyte excretion were assessed every 30 min. At 195 min, participants received an isotonic saline infusion. Secondary outcomes (vasoactive hormones, tubular transport proteins, and fluid balance) were measured at 0, 150, and 330 min.

We conducted a double‐blind, placebo‐controlled crossover trial with randomized treatment order. Participants received a beta‐hydroxybutyrate ketone monoester (KME) drink (300 mg/kg D‐ß‐hydroxybutyrate‐I‐1,3 butanediol; KE4 Pro, Ketone Aid Inc., Falls Church, VA, USA) three times per day for five days or a taste‐ and volume‐matched placebo (Ketone Aid Inc.), in randomized order. The KME drink was provided undiluted. The placebo contained purified water, organic stevia, natural flavors, potassium sorbate, and Denatonium Benzoate. After a minimum 14‐day washout period, participants were transitioned to the alternative treatment (Figure 1). On the final day of each intervention period, participants completed a 24‐h urine collection and attended examination the following day. Randomization was performed in blocks of four, and randomization, labelling, and blinding were carried out by the Department of Hospital Pharmacy, Aarhus University Hospital.

On each examination day, participants arrived in the morning after an overnight fast. They were examined in a supine position. An antecubital vein catheter was inserted in each arm. One catheter was used for blood sampling, and the other for tracer infusion. Following a 30‐min rest, the first blood samples were drawn and immediately after the first dose of KME or placebo was administered (time 0). After another 30 min, participants received an intravenous priming dose of ^99m^Technetium‐diethylene‐triamine‐pentaacetate (^99m^Tc‐DTPA), followed by a constant infusion to determine glomerular filtration rate (GFR) throughout the study day. Blood and urine samples were collected every 30 min, except for two intervals that were 45 min due to logistical constraints, until 330 min after the first dose of KME. To maintain a constant diuresis, participants received 175 mL of water every 30 min. A second dose of KME or placebo was administered 150 min after the first dose (approximately halfway through the study day). Furthermore, an infusion of 1 L of isotonic saline was administered approximately 195 min after the morning dose of KME/placebo over a 20‐min period. A brief post‐infusion pause was included due to predefined procedural requirements; therefore, the subsequent blood and urine samples were obtained 45 min after initiation of the saline infusion. Blood pressure was measured with an Omron705IT device on the arm opposite to the DTPA infusion site, immediately before and after the saline infusion and at the end of the examination day. At each time point, two measurements were taken, and the average was used for analysis.

### 2.3 End points

The primary endpoint was GFR determined by ^99m^Tc‐DTPA infusion after 5 days of KME compared with placebo, prior to the saline infusion. Secondary endpoints included fractional excretion of sodium (FENa) and potassium (FEK), urinary sodium excretion rate (UNaV), urinary potassium excretion rate (UKV), changes in systolic blood pressure and free water clearance during the examination day, as well as plasma renin and aldosterone concentrations, and urinary tubular transport proteins.

### 2.4 GFR

GFR was determined using a constant infusion technique with ^99m^Tc‐DTPA (DRN4362; Curium Pharma), as previously described (Ostergaard et al., 2021). Briefly, an intravenous priming injection of 60 MBq of ^99m^Tc‐DTPA was injected into a peripheral vein followed by a constant infusion with an individualized infusion rate based on the renal function and body weight. GFR was then calculated according to the formula:

$$\text{Urinary clearance}=\frac{\text{Urinary excretion rate}}{\text{Plasma concentration}}$$

with urinary clearance in mL/min, urinary excretion rate in cpm/min, and plasma concentration in cpm/mL.

### 2.5 FENa and FEK

Fractional excretion of sodium and potassium was calculated using the following standard formula:

$$\text{FENa}=\frac{U_{x}*V}{P_{x}*\mathrm{GFR}}*100\%$$

where *U*_*x*_ is the urinary concentration of sodium or potassium, *P*_*x*_ is the corresponding plasma concentration, and *V* is the urine output.

### 2.6 Blood‐ and urine samples

Plasma levels of aldosterone, renin and copeptin; urinary tubular transport proteins (aquaporin‐2 (AQP2) and Epithelial sodium channel γ subunit (ENaCγ)); and osmolality in plasma and urine were measured three times during the examination day: upon arrival, after 150 min, and at the end of the examination day.

Plasma levels of aldosterone, renin, and copeptin; plasma and urine osmolality; as well as urinary AQP2 and ENaCγ were measured at the University Clinic in Nephrology and Hypertension, Gødstrup Hospital, Denmark. Plasma aldosterone and renin concentrations were measured using commercially available immunoradiometric assay kits (aldosterone: R‐CW‐100; renin: KIP1531; DIAsource ImmunoAssays, Leuvain‐la‐Neuve, Belgium). Urinary AQP2 and ENaCγ were quantified using well‐established radioimmunoassays as previously described (Mose et al., 2013; Pedersen et al., 2001). Plasma and urinary osmolality were assessed by freezing point depression using an A2O osmometer (Advanced Instruments, Norwood, MA, USA). Plasma copeptin was measured with an automated immunofluorescent assay (B·R·A·H·M·S KRYPTOR Compact PLUS, ThermoFisher scientific).

Plasma concentration of β‐Hydroxybutyrate (BHB) was analyzed at the Department of Forensic Medicine, Aarhus University, using hydrophilic interaction liquid chromatography coupled with tandem mass spectrometry (HILIC‐MS/MS) (Sørensen et al., 2013).

All other routine biochemical analyses were performed at the Department of Clinical Biochemistry, Gødstrup Hospital, Denmark, according to standard laboratory procedures.

### 2.7 Study diet

During the intervention periods, a standardized diet was provided to standardize sodium and potassium intake.

Participants with an estimated daily energy requirement of up to 13,000 kJ received a diet containing 11,000 kJ per day, while those with energy requirement exceeding 13,000 kJ received 15,000 kJ per day. If participants were unable to consume the full diet, they were instructed to prioritize the main meals and foods with higher sodium content. Participants were further instructed to consume the same amount of food during both treatment periods. The macronutrient compositions were 55% carbohydrate, 15% protein, and 30% fat. The diet contained approximately 9 g of sodium chloride (154 mmol Na^+^) per day and 3.1 g or 3.7 g of potassium (≈79 or 95 mmol K^+^), corresponding to the 11,000 kJ and 15,000 kJ diets, respectively. Participants were allowed to drink only water and up to two cups of coffee or tea daily.

### 2.8 Power calculation and statistical analysis

The sample size calculation was based on the primary outcome, GFR. Assuming a mean increase of 15% and a standard deviation of 15%, a paired *t*‐test with a two‐sided significance level of 5%, 13 participants were required to detect the predefined difference under the assumption of 90% power. These estimates were based on a previous ketone infusion study in healthy males, which reported a 25% increase in GFR with a standard deviation of 10% (Fioretto et al., 1987). We chose a more conservative effect size and variability to ensure sufficient power. To allow for possible dropouts, we planned to enroll 15 participants.

All data were analyzed using R Statistical Software (v4.5.0; R Core Team 2025) using the lme4 package (version v067.i01; Bates, Maechler, Bolker and Walker) and the emmeans package (version 1.11.2‐8; Lenth). Post hoc analysis was performed using the package marginaleffects (v111.i09; Arel‐Bundock, Greifer and Heiss). All graphs were generated using the ggplot2 package (2016, Wickham). All statistical models were reviewed by a professional statistician to ensure appropriate methodology and validity.

GFR, UNaV, UKV, FENa, and FEK were analyzed using linear mixed models incorporating treatment, time, and treatment order as fixed effects and participant as a random effect. All models were assessed for a treatment × time interaction, and post hoc analyses were performed when interactions were detected. Residuals were checked for normality and homoscedasticity, and variables were log‐transformed when necessary.

We applied two separate mixed‐effects models for GFR, UNaV, UKV, FENa, and FEK corresponding to the period before (baseline) and the period after saline infusion (Figure 1). The first model was based on the plasma and urine samples collected 120–195 min after administration of the KME or placebo dose. The initial measurements following the ^99m^Tc‐DTPA injection were excluded, as urinary clearance of ^99m^Tc‐DTPA had to reach steady state before GFR could be calculated. The second mixed model included data from 195 to 330 min after morning dosing, that is, after acute sodium loading.

Vasoactive hormones, urinary tubular transport proteins, free water clearance, and plasma and urine osmolality were analyzed using a similar mixed‐model structure based on three repeated time points (in the morning before the first dose of KME/placebo, immediately before the saline infusion, and at the end of the examination day).

Outcomes from the 24‐h urine were analyzed using a paired *t*‐test.

Normally distributed data are presented as mean with the corresponding 95% confidence interval (95% CI). For log‐transformed variables, results were back‐transformed and expressed as geometric means or geometric mean ratios with 95% CI. Significance level was set at 5%.

### 2.9 Ethics

The study was approved by the Central Denmark Region Committees on Health Research Ethics (# 1‐10‐72‐67‐23). The study was registered on ClincalTrials.gov (NCT05980858) and carried out in accordance with the declaration of Helsinki 2013.

## 3 RESULTS

### 3.1 Participants

**FIGURE 2:**
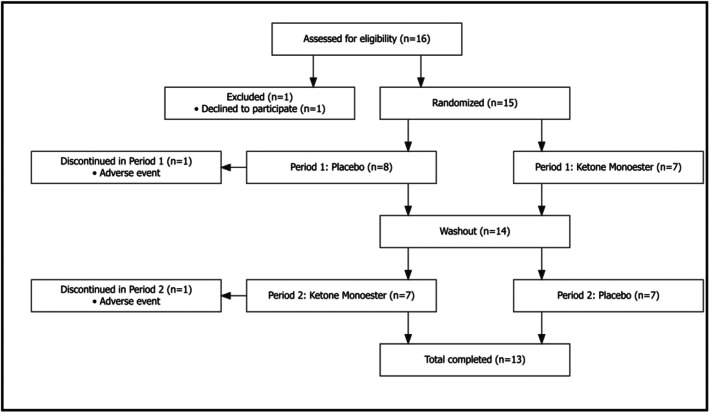
Consort diagram of participant flow through the study. A total of 16 participants were assessed for eligibility, of whom 15 were randomized. Two participants discontinued due to adverse events, resulting in 13 participants completing both study periods and being included in the final analysis.

Sixteen individuals were screened and included, and 15 were randomized. Three participants discontinued the study: one withdrew consent before randomization, and two withdrew because of adverse effects (nausea and vomiting). Of these, one discontinued during the placebo period in the first treatment sequence, and the other during the ketone period in the second treatment sequence (Figure 2). A total of 13 participants completed both study periods. Baseline characteristics are shown in Table 1. Median age was 25 years (IQR 22–28); 46% were male, and median eGFR was 109 mL/min/1.73m^2^ (IQR 103–112).

**TABLE 1:** Patient characteristics at screening.

	Analyzed: *n* = 13
Sex (male/female)	6/7
Age (years)	25 [22, 28]
BMI (kg/m^2^)	23 ± 4
Systolic BP (mmHg)	122 ± 13
Diastolic BP (mmHg)	74 ± 8
HbA1c (mmol/mol)	33 ± 2
P‐creatinine μmol/L	73 ± 15
eGFR (mL/min/1.73m^2^)	109 [103, 112]
Urinary albumin to creatinine ratio (mg/g)	6 [4, 8]

*Note*: Data are mean ± SD or median [IQR].

Abbreviations: BMI, body mass index, BP, blood pressure, eGFR, estimated glomerular filtration rate; HbA1c, hemoglobin A1c.

### 3.2 GFR and electrolyte excretion

**FIGURE 3:**
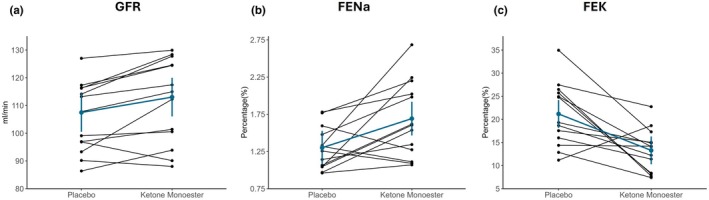
Kidney function, electrolyte excretion before saline infusion after KME treatment and placebo. Data are presented as mean ± 95% CI based on measurements from 120 to 185 min after morning dosing. (a) Glomerular filtration rate (GFR) after KME treatment and placebo (*p* = 0.002). (b) Fractional excretion of sodium (FENa) (*p* < 0.001). (c) Fractional excretion of potassium (FEK) (p < 0.001).

**FIGURE 4:**
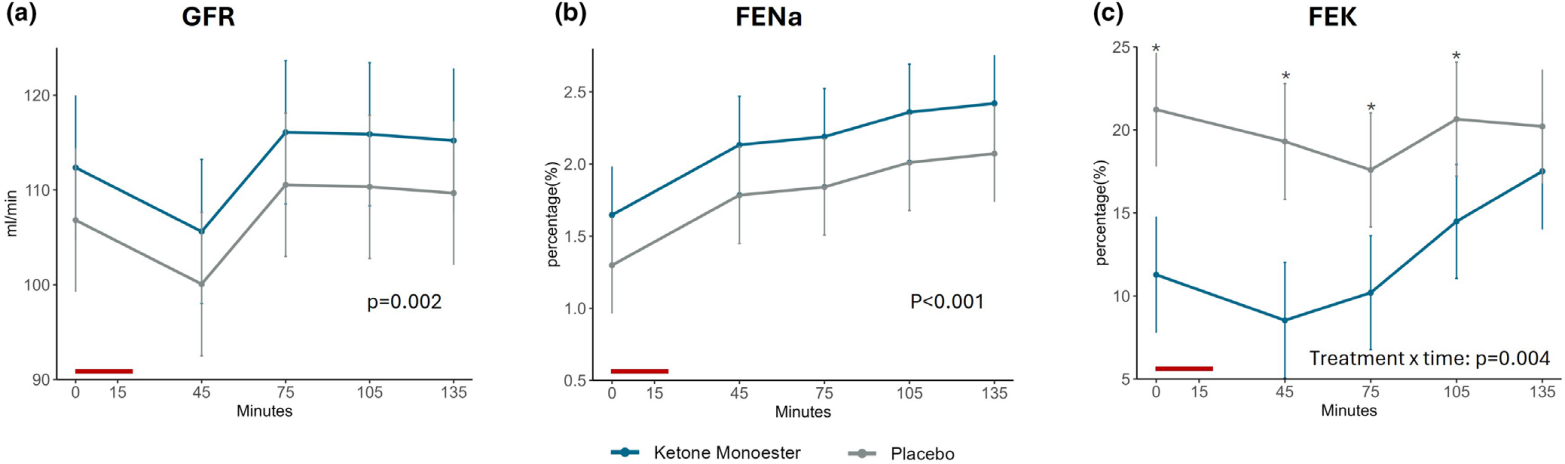
Kidney function and electrolyte handling as a function of time in minutes after saline infusion. The red line on the x‐axis indicates the duration of the saline infusion. Subsequent measurements were obtained after a predefined procedural interval (~45 min from infusion start). Data are presented as mean or geometric mean ± 95% CI. Where a treatment × time interaction was detected, post hoc analyses were performed; * indicates significant post hoc differences (*p* < 0.001 at all marked time points), whereas the final time point (135 min) was not significant (*p* = 0.103). (a) Glomerular filtration rate (GFR) in mL/min after saline infusion as a function of time. (b) Fractional excretion of sodium (FENa) in percentage after saline infusion. (c) Fractional excretion of potassium (FEK) in percentage after saline infusion.

Data are presented in Table 2. Measured GFR was higher after KME treatment compared with placebo at baseline, that is, before saline infusion on the examination day by 6.0 mL/min (95% CI 2.4, 9.7 mL/min; *p* = 0.002; Figure 3a). This effect was not influenced by the acute sodium load (*p* = 0.60, Figure 4a).

**TABLE 2:** Effects of exogenous ketosis on kidney function and electrolyte excretion.

	Ketone monoester	Placebo	Treatment × time	Mean difference (95% CI)	*p* Value
24‐h urine					
Diuresis (mL/24 h)	2184 (1769, 2739)	2289 (1920, 2687)	NA	−105 (−422, 212)	0.485
CrCl (mL/min)	141 (125, 154)	129 (120, 140)	NA	12 (−1, 25)	0.066
FENa (%)	0.41 (0.34, 0.52)	0.44 (0.36)	NA	−0.02 (−0.10, 0.05)	0.500
FE_K_ (%)*	7.8 (6.8, 8.9)	7.7 (6.7, 8.8)	NA	1.0 (0.9, 1.2)	0.784
UNaV (mmol/24 h)	115 (96, 140)	114 (98, 132)	NA	0 (−15, 16)	0.960
UKV (mmol/24 h)	64 (54, 71)	56 (50, 61)	NA	8 (−2, 18)	0.123
Baseline (before saline infusion)					
GFR (mL/min)	110 (102, 118)	104 (96, 112)	0.156	6 (2, 10)	**0.002**
Diuresis (mL/min)	6.6 (5.7, 7.4)	7.4 (6.6, 8.2)	0.147	−0.8 (−1.8, 1.2)	0.086
FENa (%)	1.7 (1.5, 1.9)	1.3 (1.0, 1.5)	0.590	0.4 (0.2, 0.6)	**<0.001**
FE_K_ (%)	13.3 (10.3, 16.3)	21.2 (18.2, 24.2)	0.184	−7.9 (−10.1, −5.7)	**<0.001**
UNaV (μmol/min)	259 (215, 303)	190 (146, 234)	0.360	69 (42, 95)	**<0.001**
UKV (μmol/min)	58 (45, 71)	89 (76, 102)	0.061	−31 (−21, −41)	**<0.001**
p‐potassium (mmol/L)	4.0 (3.9, 4.1)	4.0 (3.9, 4.1)	0.663	0.0 (0.0, 0.1)	0.207
p‐natrium (mmol/L)	140 (139, 140)	139 (139, 140)	0.157	0 (−1, 1)	0.343
After saline infusion					
GFR (mL/min)	113 (106, 120)	107 (101, 114)	0.590	6 (2, 9)	**0.001**
Diuresis (mL/min)	6.4 (5.8, 7.1)	7.2 (6.6, 7.9)	0.764	−0.8 (−1.6, 0.0)	**0.038**
FENa (%)	2.2 (1.8, 2.5)	1.8 (1.5, 2.1)	0.301	0.4 (0.2, 0.5)	**<0.001**
FE_K_ (%)	12.6 (9.5, 15.8)	19,4 (16.2, 22.6)	**0.004**	NA	NA
UNaV (mmol/min)	339 (289, 389)	270 (220, 320)	0.189	69 (44, 93)	**<0.001**
UKV (mmol/min)	56 (41, 71)	86 (71, 100)	**0.026**	NA	NA
p‐potassium (mmol/L)	4.0 (3.9, 4.1)	4.0 (3.9, 4.0)	0.957	0.0 (0.0, 0.1)	0.376
p‐natrium (mmol/L)	139 (138, 140)	140 (139, 141)	0.443	−1 (−1, 0)	**0.044**

*Note*: For 24‐h urine: values are presented as mean (95% CI) with difference as mean (95% CI). For log transformed outcomes (*) values are presented as mean (95% CI) with difference as geometric mean ratio (95% CI); *n* = 13. Comparisons were performed using paired *t*‐tests. Data from the examination day: values are presented as mean (95% CI) with difference as mean (95% CI); *n* = 13. Pairwise comparisons were performed using a mixed model with treatment, time, and treatment sequence as fixed effects, and randomization ID as a random effect. Each model was checked for treatment × time interaction. *p* values < 0.05 were considered statistically significant.

Abbreviations: CrCl, creatinine clearance; GFR, glomerular filtration rate; UO, urinary output; FE_K_, fractional excretion of potassium; FENa, fractional excretion of sodium; NA, not applicable; UKV, urinary potassium excretion rate; UNaV, urinary sodium excretion rate.

FENa and UNaV were both higher after KME treatment at baseline on the examination day (difference: 0.39%; 95% CI 0.23, 0.55%; *p* < 0.001; and 69 μmol/min; 95% CI 42, 95 μmol/min; *p* < 0.001, respectively; Figure 3b). These effects persisted after the saline infusion, and the difference between treatments was not modified by it (Figure 4c).

FEK and UKV were lower after KME treatment compared with placebo at baseline on the examination day. The mean difference for FEK was −7.9% (95% CI: −10.1, −5.7%; *p* < 0.001), and for UKV −31 μmol/min (95% CI: −41, −21 μmol/min; *p* < 0.001, Figure 3c). For both FEK and UKV, a significant effect of saline infusion was observed (*p* = 0.004 and *p* = 0.03, respectively). Potassium excretion remained lower after KME throughout the examination day, though the difference between treatments diminished over time after saline infusion (Figure 4c).

Creatinine clearance was numerically higher after 5 days of KME treatment, with a mean difference of 12 mL/min (95% CI −1 to 25 mL/min; *p* = 0.066). Sodium and potassium excretion measured in the 24‐h urine did not differ between treatments.

### 3.3 Plasma concentration of BHB

**FIGURE 5:**
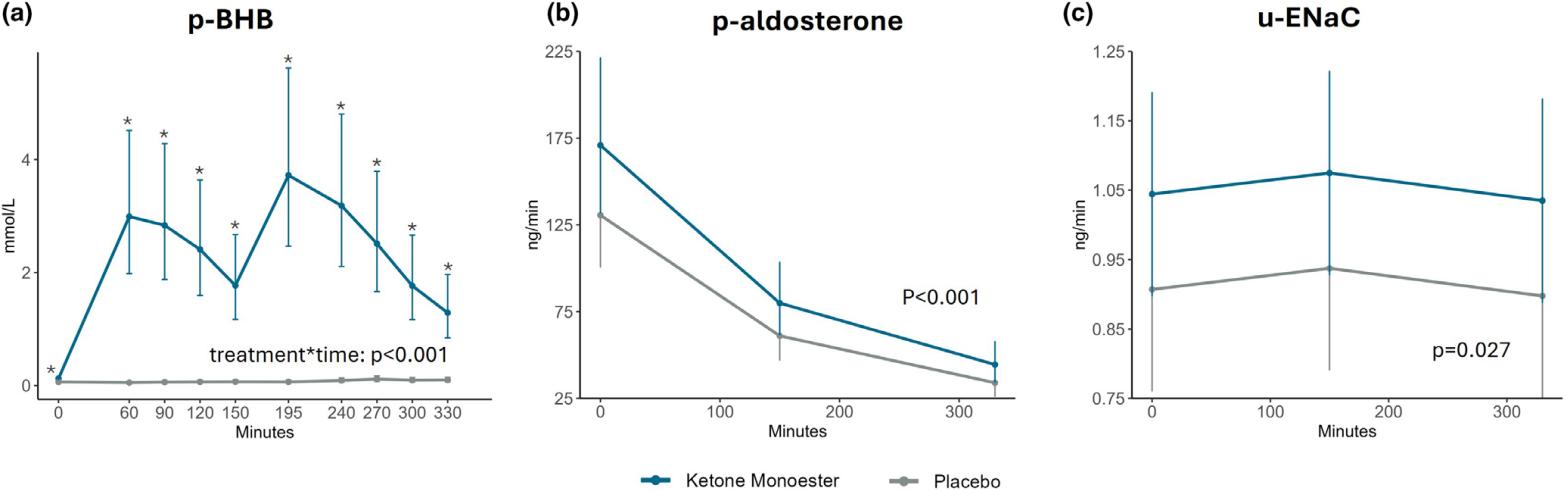
Plasma β‐hydroxybutyrate, plasma aldosterone, and urinary ENaC over time after morning dosing on the examination days. Time is shown on a continuous scale. Samples were obtained at 30‐min intervals, except for two 45‐min intervals (150–195 and 195–240 min) due to logistical reasons. Data are presented as mean or geometric mean ± 95% CI. Where a treatment × time interaction was detected, post hoc analyses were performed; * indicates significant post hoc differences (*p* < 0.001 at all marked time points except time 0, where *p* = 0.004). (a) Plasma concentration of β‐hydroxybutyrate (p‐bhb) in mmol/L. (b) Plasma concentrations of aldosterone in pg/mL. **F:** Urinary epithelial sodium channel γ subunit (u‐ENaCγ) excretion rate in ng/min.

Plasma concentrations of BHB were higher after KME treatment compared with placebo (*p* < 0.001) (Figure 5a). Morning pre‐dose concentrations were also elevated, showing a 1.97‐fold higher concentration relative to placebo (95% CI 1.11, 3.46; *p* = 0.020). The difference in plasma BHB between treatments changed over time (*p* < 0.001). In the KME arm, BHB concentrations showed two peaks, occurring approximately 30 min after each KME dose. The first peak reached a mean plasma BHB concentration of 3.0 mmol/L (95% CI 2.0, 4.4 mmol/L), and the second peak 3.7 mmol/L (95% CI 2.4, 5.6 mmol/L).

### 3.4 Vasoactive hormones and hemodynamic responses

Data are presented in Table 3. KME treatment increased plasma aldosterone levels by 30% (geometric mean ratio 1.3; 95% CI 1.1, 1.5; *p* < 0.001, Figure 5b) and aldosterone‐to‐renin ratio (ARR) by 20% (geometric mean ratio 1.2; 95% CI 1.0, 1.4; *p* = 0.023) compared with placebo. Plasma renin concentrations were numerically higher after KME treatment compared with placebo (geometric mean ratio 1.1; 95% CI 1.0, 1.2; *p* = 0.116). Aldosterone, renin, and ARR all decreased during the examination day in response to water and sodium loading.

**TABLE 3:** Vasoactive hormones, blood pressure, tubular transport proteins, and fluid balance.

	Ketone monoester	Placebo	Treatment × time	Mean difference (95% CI)	P value
Vasoactive hormones and hemodynamic parameters					
P‐aldosterone (pg/mL)*	84.7 (66.3, 108.2)	64.7 (50.7, 82.7)	0.106	1.3 (1.1, 1.5)	**<0.001**
P‐renin (pg/mL)*	6.9 (5.7, 8.4)	6.3 (5.2, 7.7)	0.364	1.1 (1.0, 1.2)	0.116
ARR*	12.2 (10.0, 14.9)	10.2 (8.4, 12.5)	0.079	1.2 (1.0, 1.4)	**0.023**
Systolic BP (mmHg)	120 (115, 126)	118 (113, 123)	0.810	2 (0, 5)	0.080
Heart rate (beats/min)	62 (57, 66)	54 (50, 59)	0.120	8 (6, 9)	**<0.001**
Tubular transport proteins and fluid balance					
Free water clearance (ml/ min)	2.1 (1.5, 2.7)	2.6 (2.0, 3.2)	0.934	−0.5 (−1.1, 0.2)	0.178
P‐copeptin (pmol/L)*	4.0 (2.9, 5.3)	3.8 (2.9, 5.2)	0.975	1.0 (0.9, 1.2)	0.487
AQP2 excretion rate (ng/min)	0.53 (0.48, 0.59)	0.51 (0.46 0.56)	0.688	0.02 (−0.01, 0.07)	0.256
ENaC excretion rate (ng/min)	1.1 (0.9, 1.2)	0.9 (0.8, 1.0)	0.153	0.10 (0.01, 0.20)	**0.027**
Plasma osmolarity (mOsm/Kg)	284 (282, 285)	284 (282, 285)	0.934	0 (−2, 2)	0.806
Urine osmolarity (mOsm/Kg)*	204 (183, 228)	176 (158, 196)	0.975	1.2 (1.0, 1.3)	0.052
Metabolic parameters					
p‐glucose (mmol/L)	4.4 (4.3, 4.5)	5.0 (4.9, 5.1)	0.140	−0.6 (−0.7, −0.5)	**<0.001**
p‐insulin (mU/L)	3.7 (3.2, 4.2)	3.5 (3.0, 4.0)	0.428	0.2 (−0.2, 0.6)	0.399

*Note*: Values are presented as mean (95% CI) with difference as mean (95% CI). For log transformed outcomes (*) values are presented as mean (95% CI) with difference as geometric mean ratio (95% CI); *n* = 13. Pairwise comparison was performed using a mixed model incorporating treatment, time and treatment sequence as fixed variables and randomization ID as random variable. Each model was checked for interaction between treatment × time. *p* values < 0.05 were considered statistically significant.

Abbreviations: ARR, aldosterone‐to‐renin ratio; AQP2, aquaporin‐2; BP, blood pressure; u‐ENaC, urinary epithelial sodium channel; u‐NCC, Na^+^‐Cl^−^ Cotransporter.

Systolic blood pressure showed no significant change but a tendency towards a small increase was found after KME treatment compared with placebo on the examination day (mean difference 2 mmHg; 95% CI 0, 5 mmHg; *p* = 0.080). Heart rate was on average 8 beats/min higher after KME treatment (95% CI 6.0, 9.0 beats/min; *p* < 0.001).

### 3.5 Tubular transport proteins and fluid balance

KME treatment led to an increase in urinary ENaCγ excretion rate compared with placebo, with a mean difference of 0.1 ng/min (95% CI 0.01, 0.2 ng/min; *p* = 0.027, Figure 5c).

At baseline, diuresis tended to be lower after KME treatment compared with placebo (−0.8 mL/min; 95% CI –1.8, 1.2 mL/min; *p* = 0.086). After the saline infusion, the estimated difference remained of similar magnitude (−0.8 mL/min) but was more precisely defined (95% CI –1.6, 0.0 mL/min; *p* = 0.038). The saline infusion did not influence the difference between treatments (*p* = 0.764). There was no difference in 24‐h diuresis between treatments (*p* = 0.485). Urine osmolality was numerically higher after KME treatment, with an estimated increase of 15% (geometric mean ratio 1.15; 95% CI 1.00, 1.34; *p* = 0.052) compared with placebo. KME treatment did not significantly affect free water clearance, plasma copeptin levels, urinary AQP2 excretion, or plasma osmolality. Data are elaborated in Table 3.

### 3.6 Metabolic parameters

Plasma glucose decreased at baseline on the examination day after KME treatment compared with placebo (mean difference: −0.6 mmol/L; 95% CI −0.7, −0.5 mmol/L; *p* < 0.001). No difference was found in insulin levels between treatments (*p* = 0.399).

## 4 DISCUSSION

Our findings show that short‐term KME treatment in healthy adults acutely increases GFR and urinary sodium excretion while reducing urinary potassium excretion. The subsequent sodium load did not alter these patterns, although the difference in potassium excretion between treatments diminished over time. These effects were evident throughout the examination day, but 24‐h sodium excretion remained unchanged on day five of supplementation, suggesting that the natriuretic response was not persistent over the full 24‐h period. Mechanistic interpretations should be considered hypothesis‐generating, as we did not directly assess tubular transporter activity or acid–base status. To our knowledge, this is the first randomized study to describe short‐term renal effects of exogenous ketosis and to characterize its glomerular and tubular consequences under controlled dietary conditions.

### 4.1 Increase in glomerular filtration rate

The observed increase in GFR is consistent with previous infusion studies demonstrating acute increases in renal plasma flow (RPF) and GFR after infusion of sodium BHB in both healthy participants and patients with type 1 diabetes (Fioretto et al., 1987; Trevisan et al., 1987). In the study by Fioretto et al., infusion of D,L‐3‐hydroxybutyric acid and its sodium salt increased RPF and GFR by 20% and 25%, respectively, in healthy subjects, and greater responses were found in patients with type 1 diabetes (Fioretto et al., 1987). Other studies similarly reported elevated RPF and GFR after acetoacetate (another ketone body) infusion (Trevisan et al., 1987). However, in contrast to our findings, increased sodium reabsorption was reported (Fioretto et al., 1987).

Beyond infusion studies, a 12‐week ketogenic dietary intervention incorporating adjunctive low‐dose KS supplementation in patients with ADPKD reported real‐world outcomes with a 6.3% increase in eGFR from baseline to the end of the intervention (Muensterman et al., 2025). However, this programme differed substantially from the present study, as ketosis resulted mainly from dietary intervention, which generally produces more sustained ketone exposure, whereas exogenous monoester supplementation induces intermittent increases in circulating BHB.

The mechanisms behind the observed GFR increase in our study remain unclear. Ketone bodies may directly affect renal function—possibly via afferent arteriolar vasodilation resulting in increased RPF and glomerular filtration (Fioretto et al., 1987; Trevisan et al., 1987). If a comparable increase in renal blood flow occurs during oral ketone monoester supplementation, this could contribute to the observed increase in GFR in the present study. Fioretto et al. proposed that the observed rise in GFR might also reflect altered tubuloglomerular feedback, as increased tubular sodium reabsorption would be expected to reduce sodium delivery to the macula densa and thereby elicit afferent vasodilation (Fioretto et al., 1987). We, however, observed enhanced sodium excretion, which would be anticipated to cause afferent vasoconstriction rather than dilation (Ichikawa, 1982). Thus, this mechanism is unlikely to explain the GFR increase observed in our study. However, these interpretations remain speculative and warrant further mechanistic investigation.

While longer‐term animal studies have suggested potential anti‐inflammatory and anti‐fibrotic effects of sustained ketosis, these mechanisms are unlikely to explain the short‐term GFR changes observed in our study (Ishimwe et al., 2020; Schreier et al., 2025).

### 4.2 Sodium excretion

The increase in sodium excretion observed after short‐term KME treatment mirrors what is seen in endogenous ketosis induced by fasting. Earlier studies have demonstrated that fasting triggers pronounced natriuresis during the first 3–5 days, accompanied by compensatory aldosterone release (Kolanowski et al., 1976). The mechanism is thought to involve increased tubular excretion of negatively charged ketone anions balanced by sodium counter‐ions (North et al., 1974; Sigler, 1975). After approximately 2 weeks of fasting or ketogenic dieting, both natriuresis and kaliuresis subside as sodium balance is restored (Skartun et al., 2025). In our study, diuresis was lower after KME treatment compared with placebo. This pattern argues against the increased sodium excretion being solely explained by an osmotic effect of ketone bodies.

Both absolute and fractional sodium excretion increased after KME treatment, indicating reduced tubular sodium reabsorption. The mechanisms by which ketosis alters tubular sodium handling remain incompletely understood. The observed increases in plasma aldosterone and urinary ENaCγ excretion after KME treatment would be expected to increase sodium reabsorption and not, as we found, decrease reabsorption (Kristensen et al., 2022). The elevations in aldosterone and ENaCγ are therefore likely to represent a compensatory response. The slight rise in blood pressure, which would typically suppress RAAS activity, further supports the interpretation that RAAS activation was compensatory rather than a primary driver of the altered electrolyte excretion. Importantly, urinary ENaCγ excretion has previously been shown to decline after hypertonic saline loading, consistent with reduced ENaC activity (Graffe et al., 2012). However, it cannot be excluded that changes in urinary ENaCγ excretion do not directly translate into proportional changes in functional channel activity.

Ketone bodies can also act as signaling metabolites. β‐Hydroxybutyrate (BHB) functions as an endogenous inhibitor of class I histone deacetylases and as a ligand at the G‐protein‐coupled receptor HCAR2 (GPR109A) (Shimazu et al., 2013; Spigoni et al., 2022). Activation of HCAR2 suppresses adenylate cyclase and reduces intracellular cyclic adenosine monophosphate (cAMP). Although these signaling pathways have not been directly linked to renal sodium transport, activation of the cAMP–PKA cascade has been shown in animal models to promote phosphorylation and membrane trafficking of key tubular transporters, including sodium–potassium–2‐chloride cotransporter (NKCC2) and ENaC (Ares et al., 2011; Kwon et al., 2013; Ortiz, 2006). NKCC2, located in the thick ascending limb of Henle's loop, reabsorbs approximately 25–30% of filtered NaCl, and its activity is stimulated by cAMP‐dependent phosphorylation and membrane trafficking (Ares et al., 2011; Ortiz, 2006). Consequently, a reduction in intracellular cAMP during ketosis could theoretically suppress NKCC2 activity and thereby reduce tubular sodium reabsorption. Because NKCC2 was not measured, any contribution of this pathway to the observed natriuresis should be considered tentative. In contrast, ENaCγ was increased, likely due to aldosterone‐mediated upregulation.

In our study, 24‐h sodium excretion remained unchanged after 5 days of ketone supplementation, suggesting that the natriuretic effect might be transient and contingent on the presence of circulating BHB. Accordingly, these changes do not translate into sustained net sodium loss over a 24‐h period within the short duration of the intervention and may therefore be of limited clinical relevance. Because participants received three daily doses, they were exposed to intermittent ketosis; however, without continuous ketone monitoring, the duration of ketosis remains uncertain. Future longer‐term studies incorporating continuous ketone monitoring are required to determine whether sustained renal effects emerge with prolonged exposure and to better characterize the temporal dynamics of these responses.

Collectively, these findings indicate that KME treatment acutely increases sodium excretion, potentially through anion‐coupled sodium loss and reduced tubular reabsorption.

### 4.3 Potassium excretion

Unlike prior fasting or ketogenic diet studies, which reported increased potassium excretion during early adaptation (Skartun et al., 2025; Veverbrants & Arky, 1969), we observed reduced absolute and fractional potassium excretion under acute exogenous ketosis. Several mechanisms may explain this novel result.

First, fundamental physiological differences exist between endogenous and exogenous ketosis. Fasting induces a catabolic state characterized by protein breakdown, low insulin levels, and increased urinary nitrogen loss (Palmer & Clegg, 2021). Insulin increases cellular potassium uptake by stimulating the Na^+^/K^+^‐ATPase (Deachapunya et al., 1999). During fasting, lower insulin availability may therefore limit Na^+^/K^+^‐ATPase–mediated potassium uptake, increasing the renal potassium load. In contrast, during exogenous ketosis insulin concentrations remain largely unchanged, and there is no presence of a catabolic state. Indeed, β‐Hydroxybutyrate infusion has previously been shown to exert anticatabolic effects (Thomsen et al., 2018). These differences between exogenous and endogenous ketosis may explain why KME treatment did not elicit the early kaliuresis characteristic of fasting.

Second, mild acidosis could have contributed to decreased potassium excretion. Ketone monoesters are metabolized to β‐hydroxybutyrate, a weak acid that transiently lowers blood pH (Poffé et al., 2021; Stubbs et al., 2017). Reductions in pH can suppress potassium secretion by inhibiting ROMK channels and the H^+^/K^+^‐ATPase in α‐intercalated cells of the collecting duct (Garg, 1991). Although pH was not measured in our study, this remains a plausible explanation for the reduced potassium excretion observed after KME treatment.

Third, modulation of tubular signaling pathways may also be involved. As previously discussed, β‐hydroxybutyrate can lower intracellular cAMP. Consequently, a reduction in NKCC2 activity would reduce the lumen‐positive transepithelial potential that drives ROMK‐mediated potassium secretion (Ares et al., 2011). As NKCC2 and ROMK activity were not measured, the involvement of this pathway remains speculative and warrants targeted mechanistic investigation.

Conversely, the presence of poorly reabsorbed anions increases the lumen‐negative transepithelial potential in the cortical collecting duct and thereby stimulates—rather than suppresses—potassium secretion (Al‐Qusairi et al., 2023). No studies have specifically examined whether ketone anions can increase the lumen‐negative transepithelial potential. In our study, the potassium content was 1.6 mg/mL in the ketone supplement and approximately 0.3 mg/mL in the placebo drink. However, the slightly higher potassium content in the KME supplement is unlikely to explain the observed differences, as a higher potassium intake would typically be expected to increase, rather than decrease, potassium excretion. Plasma sodium and potassium remained stable, indicating that systemic electrolyte disturbances were unlikely.

### 4.4 Hemodynamic responses

In the present study, ketone monoester supplementation was associated with a modest increase in heart rate, while blood pressure remained unchanged. In previous studies of exogenous ketone administration in patients with heart failure and reduced ejection fraction, increases in heart rate of comparable magnitude have been observed alongside improvements in cardiac output and stroke volume, without evidence of adverse haemodynamic effects or safety concerns (Berg‐Hansen et al., 2024; Nielsen et al., 2019).

### 4.5 Adverse events

Two participants withdrew due to gastrointestinal symptoms (nausea and vomiting), with one withdrawal occurring during the ketone monoester period and one during the placebo period. Gastrointestinal discomfort is a recognized and generally mild side effect associated with ingestion of ketone monoester supplementation (Soto‐Mota et al., 2019). No serious adverse events were reported.

### 4.6 Strengths and limitations

Key strengths of this study include its randomized, placebo‐controlled, crossover design and the use of direct GFR measurement by ^99m^Tc‐DTPA constant infusion, which yields more precise estimates than eGFR. Unlike the single‐bolus method common in clinical practice, constant infusion enables evaluation of dynamic GFR changes throughout the examination day.

Another strength was the use of a standardized diet during both treatment periods, with only water and two cups of coffee or tea permitted as fluid intake. This ensured that changes in electrolyte excretion were not confounded by dietary variation.

Limitations include the modest sample size and the short intervention period. Tubular transport was inferred indirectly from urinary excretion rates, which may not fully capture transporter activity at the epithelial surface. Nevertheless, urinary AQP2 and ENaCγ excretion have previously been validated as functional biomarkers of collecting duct transport (Graffe et al., 2012; Starklint et al., 2005). Another limitation is the 3.5‐h delay before the plasma steady state of ^99m^Tc‐DTPA, although urinary steady state occurs earlier (~1.5 h). Therefore, early measurements were excluded. Because plasma steady state had not yet been reached, GFR was estimated using the mean of two plasma concentrations obtained at the beginning and end of the urine collection period. Although this introduces minor uncertainty inherent to non–steady‐state conditions, the constant infusion method still provides far greater accuracy than creatinine‐based estimates. The study design represents a limitation in that it precludes any meaningful separation of acute examination‐day effects from those of the preceding 5‐day supplementation period. However, the first blood sample was obtained prior to the morning dose, and both aldosterone and ENaCγ excretion were consistently higher throughout the examination day without a treatment × time interaction. This suggests that the observed effects were not solely attributable to acute ketosis. Longer‐term mechanistic studies are needed to confirm these findings and to delineate the temporal pattern of renal responses to sustained exogenous ketosis.

### 4.7 Choice of ketone formulation

We used a ketone monoester to raise circulating plasma concentrations of BHB. Studies comparing KME with KS have shown that KME supplementation typically results in higher peak BHB concentrations than KS (Falkenhain et al., 2023). In addition, KS inherently deliver BHB together with inorganic cations, which may impose a substantial sodium/cation load (Fischer et al., 2018). This is relevant when interpreting renal electrolyte handling and may be undesirable in sodium‐sensitive conditions such as hypertension and chronic kidney disease. A sodium‐free ketone salt supplement (KetoCitra®) has recently been evaluated within the Ren‐Nu™ programme for autosomal dominant polycystic kidney disease as an adjunct to a ketogenic diet, delivering ~5 g/day of BHB together with potassium, calcium and magnesium (Bruen et al., 2022; Muensterman et al., 2025). In contrast, the present study investigated isolated ketone monoester supplementation in healthy individuals, resulting in ~12‐fold higher exogenous BHB delivery under controlled dietary conditions, with minimal potassium content. Concerns have been raised regarding formulation‐specific safety of chronic ketone supplementation, including preclinical reports of adverse hepatic outcomes with prolonged high‐dose ketone ester exposure (Ari & D'Agostino, 2025). Moreover, KME may induce transient reductions in blood pH, whereas KS may be mildly alkalinising due to the accompanying inorganic cations (McClure et al., 2025). However, the present study was short‐term and did not include detailed hepatic or acid–base assessments; therefore, long‐term safety and acid–base effects in patient populations should be addressed in future trials.

### 4.8 Perspectives and clinical implications

In summary, our findings indicate that short‐term exogenous ketosis elicits changes in glomerular filtration and electrolyte handling in healthy adults. This could be clinically relevant in CKD or hypertension where renal function and volume regulation are impaired. A transient increase in GFR may improve solute clearance in states of reduced filtration but could also aggravate hyperfiltration‐related injury in early CKD.

These observations suggest that ketone bodies modulate renal physiology, but the long‐term implications remain uncertain. It is not known whether these short‐term effects persist, attenuate, or reverse with sustained supplementation, nor whether they can be translated into therapeutic benefit. Studies in CKD and related patient groups are therefore needed to determine the safety profile, durability of responses, and potential clinical utility of exogenous ketosis.

## AUTHOR CONTRIBUTIONS

**Trine Z. Lyksholm:** Conceptualization; data curation; formal analysis; funding acquisition; investigation; methodology; project administration; software; validation; visualization. **Camilla L. Duus:** Validation; visualization. **Steffen F. Nielsen:** Validation; visualization. **Henrik H. Thomsen:** Conceptualization; methodology; supervision; validation. **Jesper N. Bech:** Conceptualization; funding acquisition; methodology; resources; supervision; validation; visualization.

## FUNDING INFORMATION

This research received grants from The Augustinus Foundation, the Danish Society of Nephrology, the Danish Medical Association, NIDO Research Foundation, Gødstrup Hospital, and Health Research Foundation of the Central Denmark Region. The authors received no direct funding from KetoneAid. The ketone monoester supplement and placebo were purchased using funds from the above‐mentioned grants; a standard commercial discount was applied.

## CONFLICT OF INTEREST STATEMENT

T.Z.L., C.L.D., H.H.T., and J.N.B. report no conflicts of interest, whether financial or otherwise. S.F.N. had travel expenses for a conference covered by AstraZeneca.

## Data Availability

Data will be made available upon reasonable request.
